# The accuracy of ultrasensitive PSA in predicting disease progression after radical prostatectomy

**DOI:** 10.1002/bco2.413

**Published:** 2024-09-15

**Authors:** Heikki Seikkula, Jaakko Hyysalo, Mikael Högerman, Peter J. Boström, Otto Ettala

**Affiliations:** ^1^ Department of Surgery Hospital Nova of Central Finland Jyväskylä Finland; ^2^ Department of Urology University of Turku Turku Finland; ^3^ Department of Urology Turku University Hospital Turku Finland; ^4^ Department of Mathematics and Statistics University of Turku Turku Finland

**Keywords:** biochemical recurrence, prostate‐specific antigen, prostatic neoplasms, radical prostatectomy, ultrasensitive prostate‐specific antigen

## Abstract

**Objectives:**

To assess the role of ultrasensitive PSA values (usPSA) after radical prostatectomy in predicting the subsequent biochemical recurrence (BCR).

**Material and methods:**

The study included 1836 patients who underwent open or robot‐assisted RP at Turku University Hospital between 2003 and 2018. Exclusion criteria involved patients with adjuvant treatments and those who did not reach a PSA nadir <0.1 ng/ml, resulting in a final cohort of 1313 patients. The prognostic impact of the optimal usPSA nadir cut‐off value 6 months after RP was investigated to predict subsequent BCR for the whole cohort (*N* = 1313). The optimal usPSA cut‐off value was determined for patients at 3–5 years post‐surgery (*N* = 806) and beyond 5 years (*N* = 493) of follow‐up. We used the area under the curve (AUC) calculation and the Kaplan–Meier method.

**Results:**

In a cohort with a median age of 64, primarily featuring Gleason score 7 prostate cancer. uPSA nadir of 0.01 ng/ml (AUC = 0.80) at the first monitoring post‐surgery emerged as the optimal cut‐off for identifying subjects at low (80%) or high (20%) risk of BCR within the first 3 years. Beyond this period, uPSA values during the first 3 [(AUC = 0.89; 3–5 years post‐surgery) and (AUC = 0.81; beyond 5 years)] and 5 post‐surgery years (AUC = 0.85) outperformed uPSA nadir in predicting subsequent BCR. Notably, EAU‐defined high‐risk patients with low uPSA nadir maintained substantial BCR‐free survival.

**Conclusion:**

In conclusion, a low usPSA predicts minimal BCR risk over the next 2–3 years post‐measurement. Patients with low usPSA can benefit from reduced post‐surgery PSA monitoring at 2‐ to 3‐year intervals without compromising outcomes. This strategic approach optimizes resource allocation in busy urological outpatient clinics, especially valuable in publicly reimbursed healthcare systems like Finland.

## 1 INTRODUCTION

Prostate‐specific antigen (PSA) is an important tool for monitoring patients with prostate cancer (PCa) after radical prostatectomy (RP). The American Urological Association (AUA) and European Association of Urology (EAU) define BCR after RP as repeated measurements of PSA ≥ 0.2 ng/ml.^1^, ^2^ PSA detection methods with detection levels under 0.1 ng/ml are considered ultrasensitive, and some assays are capable of detecting levels approaching 0.001 ng/ml.^3^ However, the use of ultrasensitive PSA assays (usPSAAs) remains controversial due to questions regarding the reliability and usefulness of usPSA.^2^

It has been demonstrated that detectable usPSA levels after RP can predict PCa recurrence^4^ and that usPSA could potentially detect biochemical recurrence (BCR) after RP significantly earlier than traditional PSA assays, since it improves the time to detection of BCR by months to years.^5^ This lead time to relapse seems to improve chance of durable progression‐free survival with salvage therapy given at a lower cancer burden and a wider window for cure.^6^ In addition, patients with undetectable usPSA 2 years after surgery are unlikely to develop PSADT <9 months, a risk factor for biochemical failure.^7^ However, there is no evidence that salvage RT prompted by elevated usPSA values after RP would improve patient survival.^8^

Data also show that false‐positive findings from usPSA may also originate from laboratory errors.^9^, ^10^ Previous studies have shown that the harmonization of PSA assays remained limited even after the introduction of WHO International Standards.^11^ Moreover, it has been shown that there is a substantial variation in PSA values between different PSA assays,^12^ and the comparison of usPSA values is done between different usPSAAs, the difference can occur just by variation between different methods. Thus, the same usPSAAs should be preferably used throughout the study to eliminate inter‐assay difference.

It has been proposed that the usPSA cut‐off point of 0.04 ng/ml 3 years after the operation is optimal for predicting the risk of delayed BCR.^13^ Some studies indicate that undetectable usPSA nadir after RP predicts low risk for early BCR or at least appears to confer with a favourable prognosis.^5^, ^14^ Some have even reported that undetectable usPSA nadir is a superb independent variable in predicting a favourable BCR free survival.^15^

The primary objective of this study is to assess the optimal usPSA cut‐off, which predicts subsequent biochemical progression after radical RP.

## 2 MATERIALS AND METHODS

### 2.1 Study design

A retrospective study conducted in Turku University Hospital. The study was approved by the ethics committee of the Hospital District of Southwest Finland.

### 2.2 Study population

**FIGURE 1:**
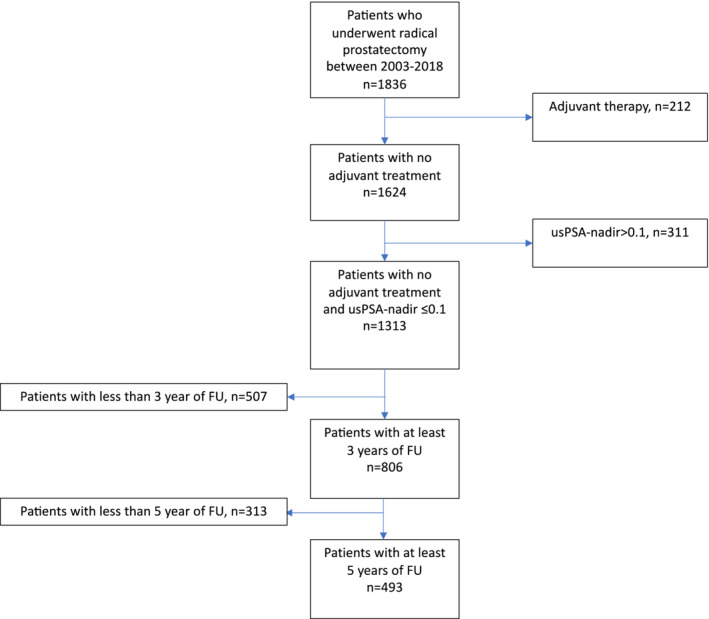
A study flowchart.

The study included a total of 1836 patients who underwent open or robot‐assisted RP at Turku University Hospital between October 2003 and December 2018. Those who received neoadjuvant, or adjuvant androgen deprivation therapy or adjuvant radiation therapy, or did not reach a PSA nadir (lowest level) of less than 0.1 ng/ml within 6 months after the operation were excluded. Additionally, a minimum of 1‐year follow‐up time was required, resulting in a total of 1313 patients included in the study (Figure 1).

### 2.3 PSA monitoring

PSA values were measured based on normal clinical practice, and no predefined surveillance strategy was used. Generally, the first PSA value, the PSA nadir, was monitored at 3–6 months post‐surgery, 12 months and yearly thereafter. BCR was defined as a PSA level greater than 0.2 ng/ml. All PSA analyses were performed using an electrochemiluminescence immunoassay (ECLIA) from Roche Diagnostics, with a detection threshold of 0.006 ng/ml.

### 2.4 Prediction of BCR based on usPSA

The predictive value of usPSA was studied at three different time points: (1) usPSA nadir, that is, at first monitoring of usPSA post‐surgery. This time point includes all 1313 subjects. (2) usPSA value during the first 3 years post‐surgery. This time point includes only subjects who have not experienced BCR during this interval, that is, usPSA has remained under 0.1. Since it is normal that usPSA fluctuates, the highest usPSA value during the first 3 years was used for the prediction. (3) usPSA value during the first 5 years post‐surgery. Details of the prediction are as in time point 2.

### 2.5 Prostate cancer risk groups

To study predictive value of usPSA in different prostate cancer EAU risk groups the population was divided as follows: low risk, PSA ≤ 20, Gleason < 8 or pathological T‐stage (pT) < 3; high risk, PSA > 20, Gleason ≥ 8 or pT ≥ 3.

### 2.6 Outcomes

Optimal usPSA‐nadir value, that is, time point 1, which predicts BCR at 3 years, at 5 years and beyond 5 years after surgery; optimal usPSA value at time point 2, which predicts BCR at 5 years and beyond 5 years after surgery; and optimal usPSA value at time point 3, which predicts BCR beyond 5 years after surgery.

### 2.7 Statistical analysis

Continues variables are presented as means (standard deviation, SD) or medians (interquartile range, IQR) depending on their normality, whereas categorical variables are presented as *n* (%). The optimal cut‐off level for usPSA to predict BCR was studied using receiver operating characteristic (ROC) calculations to maximize sensitivity and specificity. The area under the curve (AUC) and true and false rates were calculated to study the best cut‐off level. The survival figures were illustrated according to the calculated optimal usPSA cut‐off level and risk groups for PCa. The log‐rank test was used to compare subgroups for optimal usPSA level and risk levels for prostate cancer of patients in terms of BCR. The statistical significance level was set at 0.05 in all tests (two‐tailed), and 95% confidence intervals (CI) were calculated. The analyses were performed using RStudio version 1.4.1717 based on R version 4.1.

## 3 RESULTS

### 3.1 Study population

The basic characteristics of the study population are presented in Table 1. In the whole cohort, the median age was 64 (60–68) years. Most of the population had pathological Gleason score 7/ISUP grade 2–3 PCa (62%), around a quarter of the population had ISUP grade 1 (28%), and others had ISUP grade 4–5 (11%) or unknown ISUP grade PCa in the RP specimen. The rate of positive surgical margin was 27%. The proportion of patients with pT3 PCa was 500 (39%), while 793 (61%) of the patients had pT2 PCa. Subjects in the time points 2 and 3 had slightly lower PSA at pre‐surgery, final histopathological ISUP grade was more frequently GGG1, pathological T‐stage was more frequently pT3, N‐stage was less frequently pN1, M‐stage was more frequently not assessed (pMx), and surgical margin more was frequently positive compared to subjects in time point 1.

**TABLE 1:** Characteristics of the study population at three different time points.

	Time point 1 (*n* = 1313)	Time point 2 (*n* = 806)	Time point 3 (*n* = 493)
Age, median (IQR)	64.1 (59.7–68.3)	64.1 (59.6–68.2)	63.4 (59.1–67.6)
PSA, median (IQR)	7.30 (5.20–10.00)	7.20 (5.0–10.0)	7.10 (5.0–9.9)
Pathological ISUP grade/Gleason, *n* (%)			
1/2–6	362 (27.7%)	284 (35.5%)	220 (44.8%)
2/7	533 (40.8%)	293 (36.6%)	147 (29.9%)
3/7	270 (20.7%)	145 (18.1%)	83 (16.9%)
4/8	71 (5.4%)	39 (4.9%)	22 (4.5%)
5/9–10	69 (5.3%)	40 (5.0%)	19 (3.9%)
Unknown	8	5	2
pTNM classification, *n* (%)			
T2	793 (61.3%)	475 (59.2%)	273 (55.6%)
T3	500 (38.6%)	326 (40.6%)	217 (44.2%)
T4	1 (0.1%)	1 (0.1%)	1 (0.2%)
Unknown	19	4	18
Nx	391 (32.1%)	197 (25.1%)	74 (15.6%)
N0	803 (65.9%)	574 (73.1%)	392.0 (82.5%)
N1	25 (2.1%)	14 (1.8%)	9 (1.9%)
Unknown	94	21	18
M0	786 (78.4%)	432 (78.5%)	259 (87.2%)
M1	1 (0.1%)	1 (0.2%)	0 (0%)
Mx	216 (21.5%)	117 (21.3%)	38 (12.8%)
Unknown	310	256	196
Surgical margin, *n* (%)			
Negative	940 (70.8%)	568 (70.8%)	327 (66.6%)
Positive	360 (29.2%)	234 (29.2%)	164 (33.4%)
Unknown	13	4	2
BCR, *n* (%)			
No	1122 (85.5%)	703 (87.2%)	428 (86.8%)
Yes	191 (14.5%)	103 (12.8%)	65 (13.2%)

*Notes*: Time point 1: Prediction of BCR using uPSA nadir. Whole study population. Time point 2: Prediction of BCR using highest uPSA value during first 3 years post‐surgery. Subjects without biochemical recurrence during first 3 years post‐surgery. Time point 3: Prediction of BCR using highest uPSA value during first 5 years post‐surgery. Subjects without biochemical recurrence during first 5 years post‐surgery.

Abbreviations: BCR, biochemical recurrence; IQR, interquartile range; ISUP grade, International Society of Urological Pathology Grade; PSA, prostate specific antigen; pTNM classification, pathological tumour, node, and metastasis classification.

### 3.2 uPSA nadir as a prognostic factor of subsequent BCR

Table 2 demonstrates optimal usPSA values that predict BCR. During a median of 4 (IQR) years of follow‐up, a total of 191 subjects experienced BCR, of which nearly half, 88 (46%), were observed during the first 3 years, 31 (20%) during the next 2 years and 65 (34%) beyond 5 years post‐surgery. usPSA nadir (time point 1) of 0.01 ng/ml was the most optimal cut‐off to identify subjects with low (*n* = 1051, 80%) or high risk (*n* = 262, 20%) for BCR during the first 3 years post‐surgery (AUC 0.769). Hypothetically, if the cut‐off would be used to identify subjects who do not experience BCR during the first 3 years post‐surgery based on usPSA nadir lower than 0.010 ng/ml, 1024 (84%) subjects would have been correctly identified and put on less frequent usPSA surveillance, whereas only 27 (2%) subjects would have been incorrectly identified. When it comes to BCR prediction within a time frame between 3 and 5 years, and beyond 5 years after surgery, the predictive value of usPSA nadir decreases significantly, AUC 0.720 and AUC 0.659, respectively. Low usPSA values during the first 3 years post‐surgery (time point 2) and during the first 5 years (time point 3) predict subsequent BCR more accurately than usPSA nadir.

**TABLE 2:** The most optimal uPSA nadir cut‐off value, which predicts biochemical recurrence during first 3 years, first 5 years or beyond 5 years post‐surgery.

		BCR during 3 years	BCR during 5 years	BCR beyond 5 years
Time point 1	Cut‐off value	0.010	0.010	0.007
	AUC	0.796	0.720	0.659
	Sensitivity	0.693	0.556	0.534
	Specificity	0.836	0.838	0.773
	True positives	61	70	102
	False negatives	27	56	89
	False positives	201	192	255
	True negatives	1024	995	867
Time point 2	Cut‐off value		0.069	0.025
	AUC		0.888	0.812
	Sensitivity		0.867	0.757
	Specificity		0.891	0.801
	True positives		33	78
	False negatives		5	25
	False positives		84	140
	True negatives		684	563
Time point 3	Cut‐off value			0.023
	AUC			0.847
	Sensitivity			0.862
	Specificity			0.734
	True positives			56
	False negatives			9
	False positives			114
	True negatives			314

*Notes*: Time point 1: Prediction of BCR using uPSA nadir. Whole study population in included. Time point 2: Prediction of BCR using highest uPSA value during first 3 years post‐surgery. Only subjects without biochemical recurrence during first 3 years post‐surgery are included. Time point 3: Prediction of BCR using highest uPSA value during first 5 years post‐surgery. Only subjects without biochemical recurrence during first 5 years post‐surgery are included.

Abbreviations: AUC, area under the curve; BCR, biochemical recurrence; PSA, ultrasensitive prostate specific antigen level.

### 3.3 usPSA and prostate cancer risk groups

**FIGURE 2:**
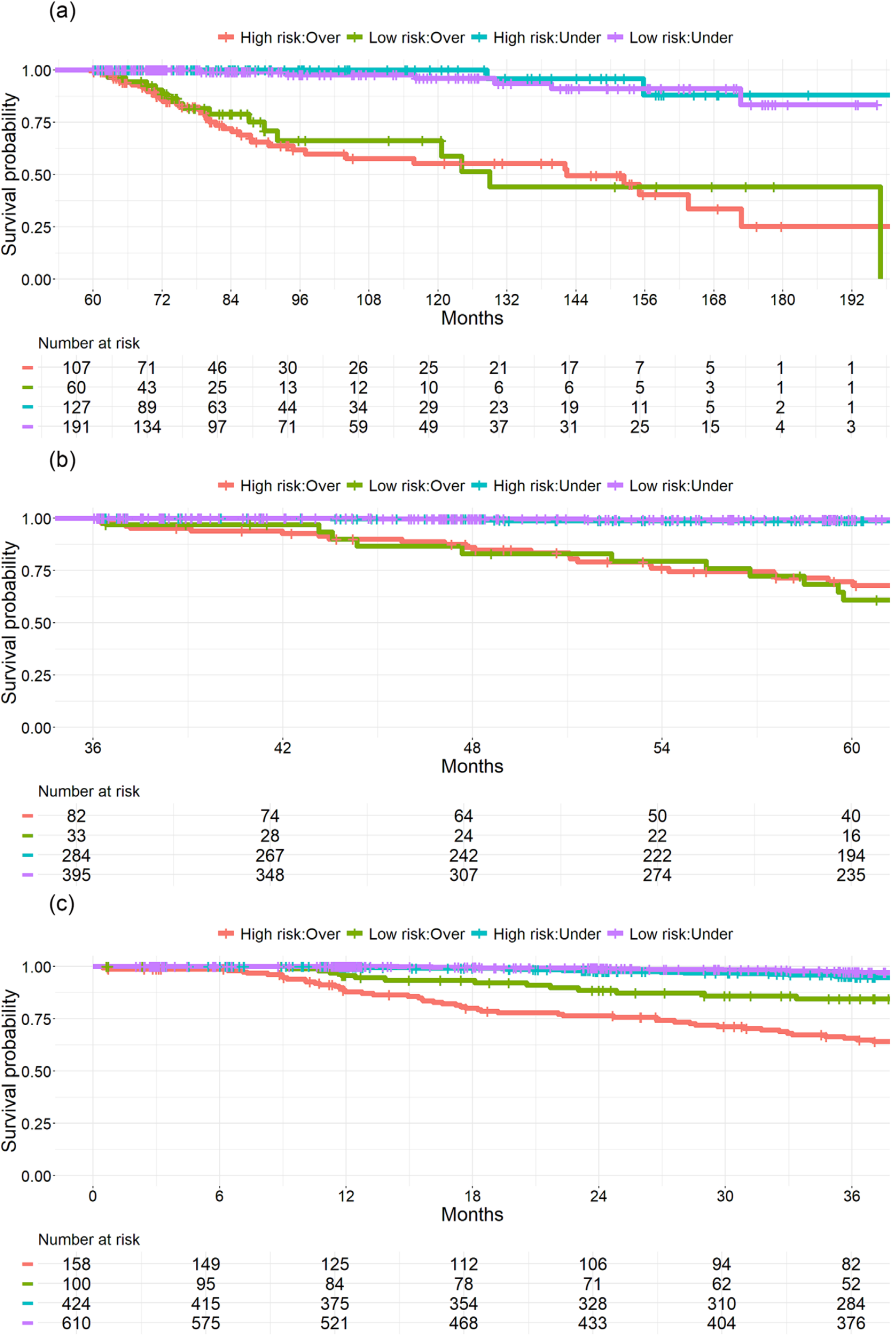
Biochemical progression free survival stratified by low usPSA levels and EAU prostate cancer risk groups. (A) Time point 1, that is, usPSA nadir. Under, usPSA nadir <0.010 ng/ml; over, usPSA nadir ≥0.010. (B) Time point 2, that is, usPSA level during first 3 years post‐surgery. Under, usPSA nadir <0.025 ng/ml; over, usPSA nadir ≥0.025. (C) Time point 3 i.e. usPSA level during first 5 years post‐surgery. Under, usPSA nadir <0.023 ng/ml; over, usPSA‐nadir ≥0.023. Prostate cancer risk group based on EAU guidelines: low risk, PSA ≤ 20, Gleason <8 or pathological T‐stage (pT) < 3; high risk, PSA > 20, Gleason ≥8 or pT ≥ 3. Lines: Red, high EAU risk and usPSA over cut‐off; green, high EAU risk and usPSA under cut‐off; blue, low EAU risk and usPSA under cut‐off; purple, low EAU risk and usPSA under cut‐off.

The probability of BCR stratified by the EAU risk group and the optimal usPSA cut‐off is depicted in Figure 2. Of the 1313 subjects, in 21 subjects, no information on EAU risk group was available, 1102 (85%) subjects were categorized as low risk and 190 (15%) as high risk. In every time point, the risk of BCR appears to be comparable between the high‐risk and lower‐risk groups when considering the nadir cut‐off values. Even patients with EAU‐defined high‐risk features demonstrated a substantial BCR‐free survival rate if their usPSA nadir remained below the defined cut‐off level.

## 4 DISCUSSION

Low usPSA nadir (time point 1) predicts subsequent BCR at best during the first 3 years after surgery, low usPSA value during the first 2 years (time point 2) at best during the time frame between 3 and 5 years post‐surgery, and low usPSA value during the first 5 years (time point 3) at best beyond 5 years post‐surgery. In addition, usPSA seems to predict BCR independently irrespectively of prostate cancer risk group.

The proposed benefit of usPSAAs after RP is that it could save high‐risk patients from unnecessary adjuvant RT and favour more selective salvage RT.^8^ Recently, full reports of three randomized clinical trials along with a pre‐planned meta‐analysis investigating the timing of RT after RP using post‐RP PSA monitoring have been published in highly ranked journals.^16^, ^17^, ^18^, ^19^ The ARTISTIC meta‐analysis was even prospectively designed before the results from the three randomized trials were known.^19^ In all three studies, adjuvant RT was compared to early salvage RT, which was triggered at a level of 0.2 ng/ml PSA in RAVES, at 0.2 ng/ml and rising in GETUG‐AFU 17 and at 0.1 ng/ml or three consecutive rises below 0.1 ng/ml in RADICALS‐RT.^16^, ^17^, ^18^ Thus, the early salvage with usPSA monitoring was only evaluated in RADICALS‐RT, the biggest of these studies, and it concluded that there was no benefit from adjuvant RT against early salvage RT.^16^ Summarizing the data, the ARTISTIC meta‐analysis indicated that there is no proven benefit from early salvage RT before PSA has reached BCR level 0.2 ng/ml.^19^

Consequently, the potential practical utility of usPSA lies in alleviating patient anxiety by indicating that ongoing PSA monitoring may no longer be imperative. Previously, similar conclusions have been made also by Malik et al., showing that a low usPSA value 3 years after the operation means low risk for biochemical recurrence also in the future.^13^ In that study regarding other PCa risk factors, in the multivariable analysis with Cox proportional hazards models, usPSA level at 3 years remained the only significant predictor of delayed BCR.^13^ Our findings substantiate the favourable accuracy of usPSA at the 3‐year post‐operation mark. Notably, in comparison to the study conducted by Malik et al., our investigation benefits from utilizing data exclusively from a single institution, employing the same usPSA assay. This advantage is particularly pertinent given the acknowledged variability in usPSA values across different PSA assay methodologies.^11^, ^13^ Furthermore, recent data shows that there is quite a remarkable variation between various PSA assays.^12^ From this standpoint, we have compelling grounds to anticipate that notable variations may similarly manifest within the usPSA range. This arises from the fact that the same assay methodologies are employed to analyse PSA levels, whether in the context of usPSA or the traditional PSA measurements.

This study successfully identified a precise usPSA nadir cut‐off point that accurately predicts the risk of biochemical progression within 6 months post‐operation. Nonetheless, the decline in PSA levels to undetectable levels post‐prostatectomy typically occurs within approximately 1 month after the operation.^20^ The decision to assess usPSA nadir at the 6‐month mark following RP was driven by the anticipation of ample time for PSA levels to exhibit a substantial decrease. This choice of a 6‐month period was made to ensure that all patients had completed their initial post‐surgery follow‐up visits. Numerous additional reports have similarly asserted that a low usPSA nadir value serves as a predictive marker for a diminished risk of early BCR.^5^, ^14^, ^15^, ^21^ Hong et al. provided evidence that a more favourable prognosis for BCR was associated with lower usPSA nadir values following RP. Their study also revealed that the area under the ROC curve, which illustrates the predictive performance of a multivariate model incorporating usPSA nadir, was significantly greater than that of the model excluding this parameter. This underscores the significance of usPSA nadir as a predictive factor for BCR and its valuable contribution to enhancing the model's predictive accuracy.^21^ Others have reported that men who attain a nadir PSA level of less than 0.01 ng/ml exhibit a notably low probability of experiencing an early relapse.^5^, ^15^ In their retrospective report, Taylor et al. contended that utilizing usPSA levels to establish the definition of BCR might not be viable. The inherent variability of PSA assays, even when operating at ultrasensitive levels, introduces substantial background noise, essentially limiting its applicability in predicting BCR. Nonetheless, their study did reach the conclusion that achieving an undetectable usPSA nadir following RP is indicative of a favourable prognosis.^14^

This study does exhibit certain limitations. A lengthier follow‐up duration would likely provide a more comprehensive assessment of the risk of delayed BCR. Considering the typically slow disease progression of PCa within the RP population, the study primarily focused on surrogate endpoints such as BCR, rather than directly estimating definitive disease progression endpoints like metastases or mortality. In our study population, we had a high proportion of patients with ISUP grades 1 and 2, which likely contributed to the relatively low number of BCR cases among the patients. Furthermore, it should be noted that patients with a PSA nadir greater than 0.1 ng/ml were excluded explaining also the rather low rate of BCR in the population.

On the other hand, the study boasts notable strengths. The substantial sample size drawn from a single academic institution ensures the availability of accurate and comprehensive data from over 1000 patients who underwent surgery. Notably, the study's use of a consistent immunoassay stands as a significant strength, eliminating variability often encountered when employing different usPSAAs.

## 5 CONCLUSIONS

In conclusion, a low usPSA value serves as an effective predictor of minimal BCR risk within the next 2 to first 3 years after the time point of measurement. Hence, patients, who display a low usPSA value, the frequency of PSA monitoring after surgery may be reduced, and monitoring PSA values at intervals of 2–3 years, while maintaining favourable patient outcomes, become feasible. This strategic approach also optimizes resource allocation within busy urological outpatient clinics, particularly valuable within publicly reimbursed healthcare systems like that in Finland.

## AUTHOR CONTRIBUTIONS

Heikki Seikkula, Peter Boström, Otto Ettala contributed to the planning of the study. Heikki Seikkula, Jaakko Hyysalo, Mikael Högeman, Otto Ettala participated in the conduction of the study. All authors contributed to the reporting of the study.

## CONFLICT OF INTEREST STATEMENT

No potential conflict of interest was reported by the authors.
